# The Effect of Glutathione on Development and Prognosis in Non-Muscle-Invasive Bladder Cancer

**DOI:** 10.3390/jcm13185483

**Published:** 2024-09-15

**Authors:** Gamze Gök, Tarık Küçük, Sertac Cimen, Alper Gök, Göksel Göktuğ, Özcan Erel, Muhammet Abdurrahim İmamoğlu

**Affiliations:** 1 Department of Biochemistry, Ankara Bilkent City Hospital, Ankara 06800, Turkey; gamze_gok@outlook.com (G.G.); erelozcan@gmail.com (Ö.E.); 2 Department of Urology, Diskapi Yildirim Beyazit Training and Research Hospital, Faculty of Medicine, University of Health Sciences, Ankara 06110, Turkey; tarikkucuk91@gmail.com (T.K.); sertaccimen@yahoo.com (S.C.); drgoksel@yahoo.com (G.G.); maimamoglu@gmail.com (M.A.İ.); 3 Department of Urology, Etlik City Hospital, Ankara 06010, Turkey

**Keywords:** bladder cancer, oxidative stress, glutathione, oxidants/antioxidants

## Abstract

**Background:** Glutathione, along with its related enzymes, constitutes a key antioxidant defense mechanism against oxidative stress and cancer formation in the body. Among urological malignancies, bladder cancer ranks second following prostate cancer. Oxidative stress has significant involvement in the development and prognosis of bladder cancer. This investigation aimed to examine the impact of glutathione on prognosis in patients with non-muscle-invasive bladder cancer. **Methods:** This study included 98 patients with high grade non-muscle-invasive bladder cancer who had undergone intravesical Bacillus Calmette–Guérin therapy and 30 healthy controls with no history of uroepithelial carcinoma of the bladder. The patients with bladder cancer were evaluated in three subgroups. Group 1 consisted of 41 patients who did not experience recurrence during follow-up, Group 2 included 28 patients who had recurrent tumors, and Group 3 consisted of 29 patients who progressed to muscle-invasive stages. Blood samples were collected from all participants. Blood levels of reduced, oxidized, and total glutathione were measured spectrophotometrically. **Results**: Reduced glutathione levels significantly differed among the groups (*p* < 0.001), attributed to the control group exhibiting higher reduced glutathione levels compared with Groups 1, 2, and 3 (*p* < 0.001). There were no significant differences in reduced glutathione levels between Groups 1 and 2, Groups 1 and 3, or Groups 2 and 3 (*p* > 0.05). Total glutathione levels varied significantly among the groups (*p* < 0.001), with the control group having higher levels than Groups 1, 2, and 3 (*p* < 0.001). No significant differences were detected between any of the paired patient groups in terms of total glutathione levels (*p* > 0.05). Regarding oxidized glutathione levels, the difference was statistically significant (*p* < 0.001), with the control group showing lower levels than the remaining three groups (*p* < 0.001). Paired comparisons revealed no significant differences in oxidized glutathione levels (*p* > 0.05). **Conclusions:** This study revealed that glutathione had an effect on the emergence of bladder cancer but did not affect its prognosis. Nevertheless, we recommend that future studies with larger bladder cancer patient cohorts should be conducted to comprehensively determine the impact of glutathione on the prognosis of this cancer.

## 1. Introduction

Glutathione (L-gamma-glutamyl-L-cysteinyl glycine) is a crucial antioxidant protecting cells against the toxic activities of peroxides, free radicals, and heavy metals. It is a tripeptide composed of cysteine, glutamic acid, and glycine. As one of the most abundant intracellular antioxidants, glutathione is predominantly found in the liver but can also be observed in all tissues and organs. Glutathione has two forms: reduced glutathione (GSH) and oxidized glutathione (GSSG), with over 90% typically in the active GSH form. An increased ratio of GSSG to GSH indicates oxidative stress [1,2,3,4]. There are different methods for measuring glutathione levels in the blood [1]. The measurement of glutathione can be performed using sophisticated devices and methods, including nanosensors, fluorometric techniques, bioluminescence, and chromatographic methods such as high-performance liquid chromatography (HPLC), liquid chromatography–mass spectrometry (LC-MS), and gas chromatography–mass spectrometry (GC-MS). Recently, Alışık et al. developed a spectrophotometric method for measuring blood glutathione. This non-enzymatic, easy-to-apply spectrophotometric method does not require sophisticated devices such as fluorometers, LC-MS, HPLC, or GC-MS. In addition, the absence of enzymes or coenzymes in this measurement method reduces costs [2]. Some enzymes play roles in maintaining glutathione activity [3,4]. For example, GSSG is converted back to GSH by the enzyme glutathione reductase [3]. Glutathione peroxidase (GPX) is an enzyme responsible for reducing hydrogen peroxide (H_2_O_2_) and organic peroxides, existing in selenium-dependent and selenium-independent forms. The selenium-dependent enzyme reduces H_2_O_2_ and lipid hydroperoxides, whereas the selenium-independent enzyme only reduces lipid hydroperoxides. To date, eight different isoenzymes of this enzyme, encoded by distinct genetic loci, have been identified (GPX 1-8) [4]. Another enzyme, glutathione-S-transferase (GSTs), is a phase 2 detoxifying enzyme responsible for the elimination of xenobiotics (environmental carcinogens, drug metabolites, chemotherapeutics, pesticides, herbicides, food preservatives, sweeteners, etc.). This enzyme also requires GSH to perform its function. GSTs catalyze the conjugation of xenobiotics with GSH, preventing damage to essential intracellular proteins and nucleic acids. Sixteen genes expressed on seven different chromosomes result in eight distinct cytosolic GSTs isoenzymes, with GST alpha, mu, pi, and theta being the most well known [5].

According to GLOBOCAN data, among urological malignancies, bladder cancer ranks second after prostate cancer, and more than 600,000 cases were diagnosed globally in 2022, with over 220,000 deaths attributed to the disease in the same year [6]. The incidence of bladder cancer varies between countries, depending on variations in risk factors and healthcare systems. Risk factors found responsible for the emergence of this cancer include tobacco smoking, dietary habits, genetic predispositions, occupational and environmental exposure to carcinogens, and oxidative stress [7]. Several studies suggest that oxidative stress has significant roles in the emergence and prognosis of bladder cancer [8,9,10,11]. For instance, in a study comparing cases of bladder cancer with healthy volunteers in relation to oxidative stress markers, namely plasma protein carbonyls, thiol groups, lipid peroxidation, and DPPH·(2,2-diphenyl-1-picrylhydrazyl) and ABTS·+ (2,2′-azino-bis (3-ethylbenzothiazoline-6-sulfonic acid)) radicals, Wigner et al. reported that all these markers were increased in the case group when compared with the controls [11]. Similarly, within the bladder cancer group itself, individuals with high-grade tumors exhibited higher levels of these markers compared with those with low-grade tumors, suggesting that oxidative stress might influence the development and prognosis of bladder cancer [11].

In the current study, we aimed to examine the impact of glutathione, one of the most important antioxidants, on the development and prognosis of non-muscle-invasive bladder cancer (NMIBC). To the authors’ knowledge, this study is the first in the literature to investigate the relationship between blood glutathione levels and prognosis in NMIBC.

## 2. Materials and Methods

Following approval from the local ethics committee (decision number: 131/09), this study included 98 patients diagnosed with NMIBC by transurethral resection (TUR) and 30 healthy individuals as a control group, all treated and monitored at the Urology Clinic of the Health Sciences University Diskapi Yildirim Beyazit Training and Research Hospital. In total, 11 (8.6%) of the participants were female and 117 (91.4%) were male. The mean age of the participants was 65.6 ± 8.5 years.

The patients with NMIBC were all those with a lamina propria-invasive histologically high-grade uroepithelial tumor (T1G3/HG) who had undergone intravesical Bacillus Calmette–Guérin (BCG) therapy using the Southwest Oncology Group protocol. Patients with variant pathologies (sarcomatoid, micropapillary, plasmacytoid variants, etc.), T1G3/HG cases with carcinoma in situ foci, and bladder cancer cases with non-uroepithelial carcinoma, such as squamous cell carcinoma and adenocarcinoma, were not included in the study. The control group was formed with healthy individuals who did not have a history of uroepithelial tumor of the bladder or any other malignancy.

The patients were divided into three groups based on whether they experienced recurrence and progression during follow-up. Group 1 consisted of 41 patients who had no recurrence or only low-grade recurrence during follow-up; Group 2 included 28 patients who experienced high-grade recurrence; and Group 3 comprised 29 patients who had progressed to the muscle-invasive stage. The control group was composed of 30 healthy volunteers.

Blood samples (3 mL) were collected from all participants to measure GSH, GSSG, and total glutathione levels. This study utilized various chemical reagents and solutions from Sigma-Aldrich, including 5,5′-Dithiobis (2-nitrobenzoic acid) (DTNB, Ellman’s reagent), methanol, ethylenediamine tetraacetic acid (EDTA), tris, hydrochloric acid (HCl), trichloroacetic acid (TCA), sodium borohydride (NaBH4), sodium hydroxide (NaOH), and sodium chloride. Measurements were conducted spectrophotometrically using a Siemens Advia 1800^®^ (Siemens Healthineers, Erlangen, Germany) device. A novel method described by Alışık et al. was employed to measure glutathione levels [2]. Samples were washed with a 0.9% NaCl solution, lysed with distilled water, and proteins were precipitated using a 20% TCA solution. TCA-treated samples were reduced with NaBH4 and NaOH, and the pH was adjusted using NaOH. Excess NaBH4 was neutralized with HCl to prevent further reduction of DTNB molecules and reoxidation of glutathione. NaBH4 was used as a reductant. After the reduction process was completed, an HCl solution was added to remove the remaining NaBH4 to prevent further reduction of DTNB molecules and re-oxidation of glutathione molecules. Glutathione levels were measured using the Ellman method, modified by Hu, with a 500 mM tris solution (pH: 8.2). In this method, the thiol residues of glutathione reduced DTNB molecules to 2-nitro-5-benzoic acid. Glutathione levels were measured before and after the reduction process. The GSSG levels were calculated by subtracting the GSH levels from the total measured glutathione levels and then dividing the result by two.

### Statistical Analysis

Data were analyzed by IBM SPSS Statistics *ver.* 25 (IBM Corporation, Armonk, NY, USA) package program. Kolmogorov–Smirnov and Levene’s tests were used to investigate whether the assumptions of the normality of data distribution and homogeneity of variances were met. Categorical data were expressed as numbers (*n*) and percentages (%), while the quantitative data were given as mean ± SD or median (25th–75th) percentiles; where appropriate. The mean differences in ages among groups were analyzed with one-way ANOVA. The Fisher–Freeman–Halton test was applied for the comparison of the gender distribution. The Kruskal–Wallis test was employed to determine the significance of differences in GSH, GSSG, total glutathione, and GSSG to GSH ratio among groups. When the *p*-values from the Kruskal–Wallis test statistics were statistically significant, a Dunn–Bonferroni multiple comparison test was used to know which groups differ from which. Multinominal logistic regression analyses were performed in order to determine the prognostic contribution of biochemical measurements after adjustment for age and gender. Adjusted odds ratios, 95% confidence intervals, and Wald statistics for each biochemical measurement were also calculated. Statistical significance level was set at less than 0.05 for each 2-tailed hypothesis testing.

## 3. Results

**Figure 1:**
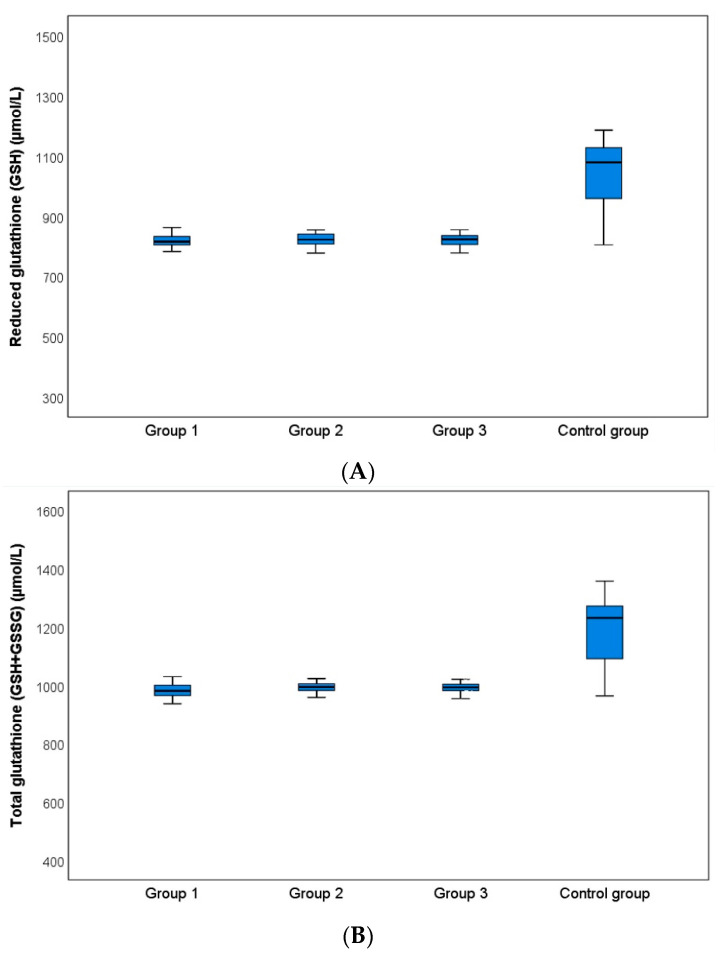

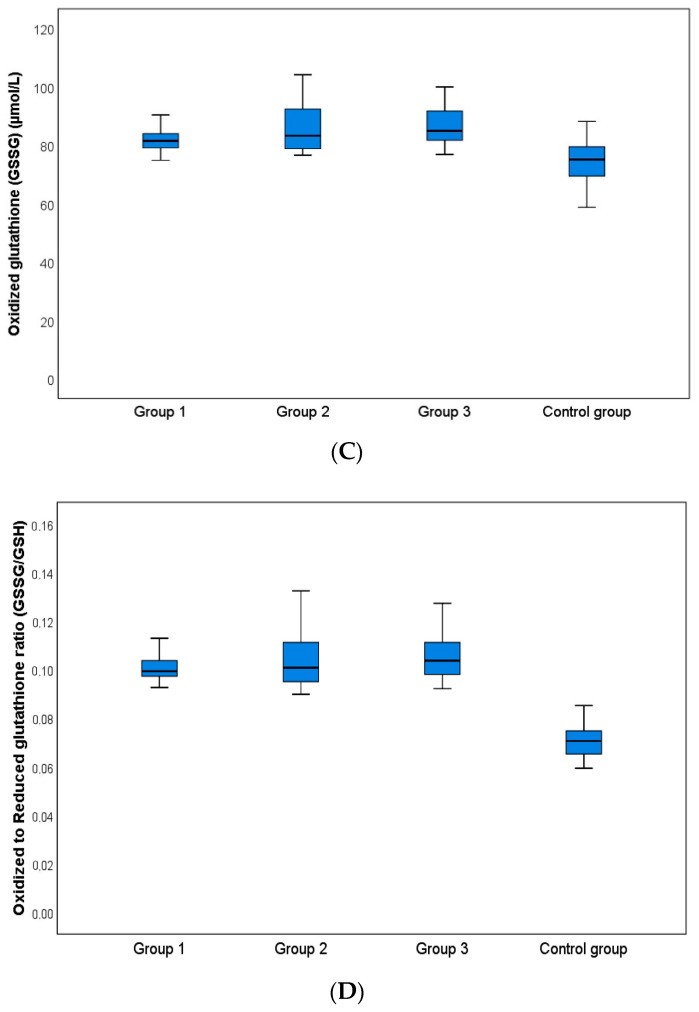
Comparison of (**A**) reduced glutathione (i.e., GSH), (**B**) total glutathione (i.e., GSH + GSSG), (**C**) oxidized glutathione (i.e., GSSG), and (**D**) oxidized glutathione to reduced glutathione ratio (i.e., GSSG/GSH) levels among groups.

Statistical analysis revealed no significant differences between the groups in terms of the mean age or gender distribution (*p* = 0.310 and *p* = 0.376, respectively). While GSH and total glutathione levels were statistically significantly lower in Groups 1, 2, and 3, respectively, compared with the control group (*p* < 0.001), otherwise GSSG and GSSG:GSH ratios were statistically significantly higher in Groups 1, 2, and 3, respectively, compared with the control group (*p* < 0.001) (See Table 1). However, there were no significant differences between Groups 1 and 2, Groups 1 and 3, or Groups 2 and 3 in terms of biochemical measurements as mentioned above (*p* > 0.05) (See Figure 1A–D).

When the group of patients without recurrence (i.e., Group 1) was selected as the reference, it was observed that GSH, total glutathione, GSSG, and the ratio of GSSG to GSH did not have any statistically significant determinant on the occurrence of recurrence (i.e., Group 2) and muscle-invasive progression (i.e., Group 3), respectively (*p* > 0.05). Similarly, when Group 2 was selected as the reference, GSH, total glutathione, GSSG, and the ratio of GSSG to GSH, respectively, did not have any statistically significant determinants on muscle-invasive progression (*p* > 0.05). In other words, these biochemical measurements were not found to have any statistically significant predictive value on prognosis (See Table 2).

## 4. Discussion

Bladder cancer is a prevalent urogenital malignancy, mostly presenting as NMIBC at initial diagnosis. Based on recurrence and progression probabilities, NMIBC can be stratified into three risk categories: low, intermediate, and high. For patients with high-risk NMIBC characterized by T1G3/HG pathology, adjuvant intravesical BCG therapy is the standard to improve prognosis. However, intravesical BCG can lead to severe adverse effects and, occasionally, mortality. Despite BCG therapy, which is associated with serious side effects, the majority of patients still experience tumor recurrence, and a significant portion progress to more advanced tumor stages. Interestingly, patients initially diagnosed with NMIBC who show progression to the muscle-invasive stage during follow-up have a poorer prognosis and survival rate than those diagnosed with muscle-invasive bladder cancer at the time of diagnosis [12]. This underscores the importance of identifying new predictive factors for determining the prognosis in high-risk NMIBC patients. Currently, clinical and pathological data, such as concurrent CIS, T category, tumor grade, gender, age, tumor count and diameter, and previous recurrence, are utilized for the assessment of NMIBC prognosis. However, it is clear that the development of new markers in addition to these clinicopathological factors is necessary to enhance the accuracy of prognostic prediction.

Given the bladder’s role as a reservoir for urine, it is exposed to oxidative waste for prolonged periods. Publications indicate significant roles of antioxidant mechanisms in the development and prognosis of bladder cancer [8,9,10,11]. Glutathione and related enzymes constitute one of the body’s most crucial antioxidant defense systems. Several studies have explored the effects of these enzymes and their genetic variants on bladder cancer prognosis [10,13,14]. For instance, in a study of 112 patients with bladder cancer, Minato et al. demonstrated that decreased GPX2 expression detected by immunohistochemistry in pathology specimens might be associated with cancer invasion [10]. Another study involving 7236 patients with bladder cancer identified that the GSTP1 gene 313 A/G (rs1695) polymorphism increased the risk of bladder cancer development [14]. A study reported by MD Anderson Cancer Center investigated the effects of various glutathione pathway genes on the prognosis following NMIBC treatment (TUR alone or TUR + BCG) [13]. In that study, 414 patients with NMIBC were individually examined for 114 single nucleotide polymorphisms (SNPs) in 21 glutathione pathway genes, and it was found that 7 SNPs were associated with recurrence in the TUR alone group, and 15 SNPs were associated with recurrence in the TUR + BCG group. The likelihood of recurrence was observed to be higher as the quantity of unfavorable genotypes increased in the glutathione pathway in both groups [13]. In another study, the genotype of the leucine to proline polymorphism located in codon 198 at GPX1 was determined by PCR in cases of bladder cancer and healthy individuals. The study concluded that the proline/leucine genotype of GPX1 was correlated with a higher risk of developing bladder cancer when compared with the proline/proline genotype [15]. Furthermore, within the bladder cancer group, the proline/leucine genotype of GPX1 was more prevalent in cases with stage T2-4 tumors compared with Ta-1 tumors, indicating its association with advanced tumor stages [15]. Therefore, the Leucin allele in GPX1 was found to be a risk allele both for the development of bladder cancer and for having advanced tumor stages in those who developed bladder cancer [15]. Albarakati et al. investigated the effect of genetic variants of phase 2 detoxifying enzymes (GSTs) on the prognosis of bladder cancer [16]. Tissue samples obtained during bladder resection were used to harvest genomic DNA in 93 cases of bladder cancer. The extracted samples were analyzed, revealing that the GSTM1-null genotype and GSTP1 (AG/GG) genotype were significantly related to poor overall survival [16]. These studies underscore the significant role of glutathione metabolism-related enzymes and their genetic variants in the development of bladder cancer.

Despite existing studies on the effects of enzymes responsible for glutathione activity and their genetic variants on bladder cancer, research directly examining the impact of glutathione itself on bladder cancer is limited. One such study by Güneş et al. measured serum GSH levels in cases of bladder cancer and healthy individuals, finding significantly lower GSH levels in the former, suggesting the involvement of oxidative stress in the etiopathogenesis of bladder cancer [17]. In another study, Mohandas et al. measured glutathione and glutathione-related enzyme levels in rabbit bladder tissues and suggested that low tissue levels of glutathione and related enzymes might predispose the bladder to cancer development induced by chemical carcinogens [18]. However, these studies did not assess the impact of glutathione on prognosis in bladder cancer [17,18]. To the authors’ knowledge, the current investigation evaluated the relationship between GSH, GSSG, and total glutathione levels in blood and the prognosis of NMIBC for the first time in the literature. In our study, the observation that healthy volunteers had higher levels of GSH and total glutathione, along with lower levels of oxidized glutathione compared with all NMIBC subgroups, suggests that glutathione may be a contributing factor in the etiopathogenesis of bladder cancer. However, the lack of significant differences in glutathione levels among the NMIBC subgroups with distinct prognostic characteristics (non-recurrent, high-grade recurrent, and progressive) indicates that glutathione is unlikely to be a determining factor in the prognosis of bladder cancer. Thus, our study suggests that disruption in the GSSG/GSH balance, a marker of oxidative stress, might be involved in the etiopathogenesis of bladder cancer but does not impact its prognosis. Our study has certain limitations, including the small size of the cohort and the timing of blood sample collection not coinciding with initial diagnosis in patients with bladder cancer. Therefore, there is a need for further studies with a prospective design and larger case series to better elucidate the impact of glutathione on prognosis in bladder cancer.

## 5. Conclusions

Identification of new markers for predicting the prognosis of bladder cancer is crucial. This study revealed that glutathione, one of the most important antioxidants, had an effect on the emergence of bladder cancer but did not affect its prognosis. Nevertheless, we recommend that future studies with larger bladder cancer patient cohorts be conducted to comprehensively determine the effect of glutathione on the prognosis of bladder cancer.

## Figures and Tables

**Table 1:** Demographic characteristics and biochemical measurements regarding study groups.

	Group 1 (*n* = 41)	Group 2 (*n* = 28)	Group 3 (*n* = 29)	Control Group (*n* = 30)	*p*-Value
Age (years)	66.1 ± 7.3	67.4 ± 8.1	65.8 ± 9.7	63.3 ± 9.0	0.310 †
Gender					0.376 ‡
Male	39 (95.1%)	26 (92.9%)	24 (82.8%)	28 (93.3%)	
Female	2 (4.9%)	2 (7.1%)	5 (17.2%)	2 (6.7%)	
Reduced glutathione (GSH) (µmol/L)	815.5 (804.8–833.5) ^a^	822.3 (807.8–841.0) ^b^	822.9 (806.5–835.5) ^c^	1079.1 (947.5–1128.1) ^a,b,c^	<0.001 ¶
Total glutathione (GSH + GSSG) (µmol/L)	980.5 (965.1–1000.8) ^a^	994.2 (980.5–1005.6) ^b^	993.0 (980.2–1005.6) ^c^	1231.8 (1088.0–1274.1) ^a,b,c^	<0.001 ¶
Oxidized glutathione (GSSG) (µmol/L)	81.5 (78.8–84.2) ^a^	83.2 (78.8–92.8) ^b^	84.9 (81.6–91.8) ^c^	75.2 (69.2–79.7) ^a,b,c^	<0.001 ¶
Oxidized/reduced glutathione (GSSG/GSH)	0.099 (0.097–0.104) ^a^	0.101 (0.095–0.112) ^b^	0.104 (0.098–0.111) ^c^	0.071 (0.065–0.754) ^a,b,c^	<0.001 ¶

Data are displayed as mean ± SD or median (25th–75th) percentiles where appropriate. †—one-way ANOVA, ‡—Fisher–Freeman–Halton test, ¶—Kruskal–Wallis test. ^a^ Group 1 vs. Control group (*p* < 0.001), ^b^ Group 2 vs. Control group (*p* < 0.001), ^c^ Group 3 vs. Control group (*p* < 0.001).

**Table 2:** The investigation of the prognostic contribution regarding biochemical measurements and the results of multinominal logistic regression analyses.

	OR (95% CI) *	Wald	*p*-Value
Group 1 vs. 2			
Reduced glutathione (GSH) (µmol/L) **	0.883 (0.701–1.113)	1.103	0.294
Total glutathione (GSH + GSSG) (µmol/L) **	0.821 (0.673–1.002)	3.767	0.052
Oxidized glutathione (GSSG) (µmol/L) ***	1.062 (0.993–1.135)	3.099	0.078
Oxidized/reduced glutathione (GSSG/GSH) ****	1.040 (0.989–1.094)	2.295	0.130
Group 1 was considered the reference group			
Group 1 vs. 3			
Reduced glutathione (GSH) (µmol/L) **	0.897 (0.710–1.133)	0.831	0.362
Total glutathione (GSH + GSSG) (µmol/L) **	0.838 (0.685–1.025)	2.959	0.085
Oxidized glutathione (GSSG) (µmol/L) ***	1.057 (0.988–1.132)	2.565	0.109
Oxidized/reduced glutathione (GSSG/GSH) ****	1.037 (0.984–1.092)	1.869	0.172
Group 1 was considered the reference group			
Group 2 vs. 3			
Reduced glutathione (GSH) (µmol/L) **	1.015 (0.794–1.298)	0.015	0.902
Total glutathione (GSH + GSSG) (µmol/L) **	1.021 (0.846–1.232)	0.045	0.832
Oxidized glutathione (GSSG) (µmol/L) ***	0.996 (0.939–1.056)	0.018	0.892
Oxidized/reduced glutathione (GSSG/GSH) ****	0.997 (0.951–1.046)	0.013	0.910
Group 2 was considered the reference group			

OR—odds ratio, CI—confidence interval. * After adjustment for age and gender. ** Effect of each 10 µmol/L decrease on prognosis. *** Effect of each 1 µmol/L increase on prognosis. **** Effect of each 0.001 unit increase on prognosis.

## Data Availability

The data that support the findings of this study are available from the corresponding author upon request.
